# Supplementary material for: Interventions for incarcerated adults with opioid use disorder in the United States: A systematic review with a focus on social determinants of health
Source: PLoS One. 2020 Jan 21;15(1):e0227968. doi: 10.1371/journal.pone.0227968 (PMC6974320; doi:10.1371/journal.pone.0227968)
Supplement: S1 Table — Completed PRISMA Checklist. (DOC) [file pone.0227968.s001.doc]

| **Section/topic** | **#** | **Checklist item** | **Reported on page #** |
| --- | --- | --- | --- |
| **TITLE** | | |  |
| Title | 1 | Identify the report as a systematic review, meta-analysis, or both. | 1 |
| Excerpt: |  | *Interventions for incarcerated adults with opioid use disorder in the United States: A systematic review with a focus on social determinants of health* |  |
| **ABSTRACT** | | |  |
| Structured summary | 2 | Provide a structured summary including, as applicable: background; objectives; data sources; study eligibility criteria, participants, and interventions; study appraisal and synthesis methods; results; limitations; conclusions and implications of key findings; systematic review registration number. | 2 |
| Excerpt: |  | *ABSTRACT*  *Incarceration poses significant health risks for people involved in the criminal justice system. As the world’s leader in incarceration, the United States incarcerated population is at higher risk for infectious diseases, mental illness, and substance use disorder…*  *…* *This review suggests an evidence gap; evidence-based interventions that address OUD and SDOH in the context of criminal justice involvement are urgently needed.* |  |
| **INTRODUCTION** | | |  |
| Rationale | 3 | Describe the rationale for the review in the context of what is already known. | 3-4 |
| Excerpt |  | *In the United States, the prison incarceration rate is the highest in the world at 655 per 100,000 [1]. Incarceration poses significant health risks for people involved in the criminal justice system [2–5]. Compared with the general population, incarcerated populations have much higher burdens of infectious diseases (e.g., hepatitis C virus, HIV, and tuberculosis) as well as mental illness and substance use disorder [6–10]. The transition from incarceration to the community itself is especially perilous [2,11,12]…* *Increased risk of overdose post-release may be explained, at least in part, by decreased drug tolerance from a reduction in use or abstinence during incarceration. Returning to drug use following release may then be fatal due to the decreased tolerance level [3]…* *Beyond MOUD treatment itself, social determinants of health (SDOH) are critical elements related to health outcomes post-release [23–25]. SDOH, as defined by the World Health Organization, are non-clinical factors including the “conditions in which people are born, grow, live, work and age. These circumstances are shaped by the distribution of money, power and resources at global, national, and local levels.” [26]. Examples include housing, transportation, socioeconomic status. Addressing SDOH and attaining health care are often interrelated difficulties and conflicting priorities for formerly incarcerated people [23–25, 27–29].* |  |
| Objectives | 4 | Provide an explicit statement of questions being addressed with reference to participants, interventions, comparisons, outcomes, and study design (PICOS). | 4-5 |
| Excerpts |  | *The purpose of this systematic review is to 1) identify interventions for OUD that have been implemented as part of criminal justice system involvement, 2) determine which interventions also include a social determinants component, and 3) note any common elements between interventions with significant outcomes.* |  |

| **Section/topic** | **#** | **Checklist item** | **Reported on page #** |
| --- | --- | --- | --- |
| **METHODS** | | |  |
| Protocol and registration | 5 | Indicate if a review protocol exists, if and where it can be accessed (e.g., Web address), and, if available, provide registration information including registration number. | 5 |
| Excerpt |  | *A formal protocol for this review can be found at dx.doi.org/10.17504/protocols.io.69zhh76.* |  |
| Eligibility criteria | 6 | Specify study characteristics (e.g., PICOS, length of follow-up) and report characteristics (e.g., years considered, language, publication status) used as criteria for eligibility, giving rationale. | 5,6 |
| Excerpt |  | *We completed a preliminary screen by removing duplicates and excluding articles that were not published in the last five years, were not published in English, did not have the full article text available, or did not include adults 19-years-old and older…* *Next, we conducted a title and abstract screen to determine if publications fell within the inclusion criteria: 1) studies conducted in the U.S., 2) intervention studies only, 3) intervention studies for OUD, 4) for adults ages 19 and older…* *We excluded publications if: they described interventional studies that were conducted outside of the United States; the population of interest was under the age of 19; if studies were not interventional (e.g. epidemiological or surveillance studies); or did not investigate primary outcomes of interest…* |  |
| Information sources | 7 | Describe all information sources (e.g., databases with dates of coverage, contact with study authors to identify additional studies) in the search and date last searched. | 5,6 |
| Excerpt |  | *We conducted a search of academic literature on May 6, 2019 to identify interventions for people with OUD implemented during incarceration following PRISMA standards for systematic reviews [34]... We used PubMed to identify peer-reviewed articles…Grey literature and contact with study authors for additional studies were not pursued as part of this review.* |  |
| Search | 8 | Present full electronic search strategy for at least one database, including any limits used, such that it could be repeated. | 5-6 |
| Excerpt |  | *We conducted all searches using a Boolean keyword search ((substance use OR medically assisted treatment OR opioid OR drug) AND (incarceration OR prison OR reentry OR jail)) in PubMed using the “best match” function. We completed a preliminary screen by removing duplicates and excluding articles that were not published in the last five years, were not published in English, did not have the full article text available, or did not include adults 19-years-old and older. We also searched ProQuest and Google Scholar using the same search terms and criteria. Publications identified using those methods were duplicates of the PubMed search and thus removed.* |  |
| Study selection | 9 | State the process for selecting studies (i.e., screening, eligibility, included in systematic review, and, if applicable, included in the meta-analysis). | 5-7 |
| Excerpt |  | *We completed a preliminary screen by removing duplicates and excluding articles that were not published in the last five years, were not published in English, did not have the full article text available, or did not include adults 19-years-old and older…Next, we conducted a title and abstract screen to determine if publications fell within the inclusion criteria: 1) studies conducted in the U.S., 2) intervention studies only, 3) intervention studies for OUD, 4) for adults ages 19 and older. Publications were excluded if: they described interventional studies that were conducted outside of the United States; the population of interest was adolescents, youth, in juvenile detention centers, or under the age of 19; if studies were not interventional (e.g. epidemiological or surveillance studies); or did not investigate primary outcomes of interest. Primary outcomes of interest include: opioid-related mortality, non-fatal overdose, post-release opiate use (heroin, injection drugs, prescription drugs), treatment initiation during incarceration, treatment initiation in community, adherence to treatment post-release, maintaining treatment post-release (i.e. keeping and attending appointments for medication treatment, continuous engagement), and withdrawal symptoms.* |  |

| **Section/topic** | **#** | **Checklist item** | **Reported on page #** |
| --- | --- | --- | --- |
| Data collection process | 10 | Describe method of data extraction from reports (e.g., piloted forms, independently, in duplicate) and any processes for obtaining and confirming data from investigators. | 6 |
| Excerpt |  | *For the publications included in final review, the data were extracted individually by investigators and then compared. Findings were compiled in a categorical matrix (Table 1).* |  |
| Data items | 11 | List and define all variables for which data were sought (e.g., PICOS, funding sources) and any assumptions and simplifications made. | 6,7 |
| Excerpt |  | *Extracted data include: study and intervention characteristics, including target population, state, sample size, time of intervention implementation (intake, post-release, civil commitment, during incarceration, post-release, pre-release), implementation setting (jail, civil commitment facility, prison, transitions clinic), study design (case report, chart review, cohort, pilot study, randomized control trial), type of opioid intervention (buprenorphine, methadone, withdrawal management, XR-NTX, patient navigation, cross-sector collaboration), comparator, whether and how SDOH were addressed in the intervention (e.g. support for housing, transportation, financing medical care, nutrition services, and case management or social services referral to navigate SDOH issues), and study outcomes.* |  |
| Risk of bias in individual studies | 12 | Describe methods used for assessing risk of bias of individual studies (including specification of whether this was done at the study or outcome level), and how this information is to be used in any data synthesis. | NA |
| Summary measures | 13 | State the principal summary measures (e.g., risk ratio, difference in means). | NA |
| Synthesis of results | 14 | Describe the methods of handling data and combining results of studies, if done, including measures of consistency (e.g., I^2^) for each meta-analysis. | NA |

| Risk of bias across studies | 15 | Specify any assessment of risk of bias that may affect the cumulative evidence (e.g., publication bias, selective reporting within studies). | 17 |
| --- | --- | --- | --- |
| Excerpt |  | *We may not have identified some pilot programs initiated by county, state, or federal departments of corrections, health departments, or community organizations because we searched only the academic literature. This review does not include programs currently implemented by respective criminal justice systems or facilities. Some existing interventions may not have publicly available evaluations. Further, carceral facilities and systems can vary significantly, even within the same county or state and so studies may not be generalizable to other settings.* |  |
| Additional analyses | 16 | Describe methods of additional analyses (e.g., sensitivity or subgroup analyses, meta-regression), if done, indicating which were pre-specified. | NA |

| Section/topic | # | Checklist item | Reported on page # |
| --- | --- | --- | --- |
| **RESULTS** | | |  |
| Study selection | 17 | Give numbers of studies screened, assessed for eligibility, and included in the review, with reasons for exclusions at each stage, ideally with a flow diagram. | 6,7 |
| Excerpt |  | *In the initial keyword search in PubMed, 6,604 citations were identified. After applying filters, 993 publications met the preliminary screen. From those, we identified 45 full-text articles through the abstract and title screen. Finally, through full review, we identified 13 publications that met all inclusion criteria (Fig 1).* |  |
| Study characteristics | 18 | For each study, present characteristics for which data were extracted (e.g., study size, PICOS, follow-up period) and provide the citations. | 7-14 |
| Excerpt |  | *Of the 32 publications removed from consideration, 14 were removed because they described studies that were not interventions, six were not implemented as part of criminal justice involvement, seven were not opioid-specific, one was not exclusively for people who are involved in the criminal justice system, and three were removed because the outcomes measured did not meet inclusion criteria. Fig 1 provides additional details in a PRISMA diagram. Of the 13 publications included for final synthesis, some included continuation studies, leaving 12 distinct interventions.* |  |
| Risk of bias within studies | 19 | Present data on risk of bias of each study and, if available, any outcome level assessment (see item 12). | NA |
| Results of individual studies | 20 | For all outcomes considered (benefits or harms), present, for each study: (a) simple summary data for each intervention group (b) effect estimates and confidence intervals, ideally with a forest plot. | 9-12 |
| Excerpt |  | *Results are described in Table 1 and tabulated in Table 2.* |  |
| Synthesis of results | 21 | Present results of each meta-analysis done, including confidence intervals and measures of consistency. | NA |
| Risk of bias across studies | 22 | Present results of any assessment of risk of bias across studies (see Item 15). | NA |
| Additional analysis | 23 | Give results of additional analyses, if done (e.g., sensitivity or subgroup analyses, meta-regression [see Item 16]). | NA |

| Section/topic | # | Checklist item | Reported on page # |
| --- | --- | --- | --- |
| **DISCUSSION** | | |  |
| Summary of evidence | 24 | Summarize the main findings including the strength of evidence for each main outcome; consider their relevance to key groups (e.g., healthcare providers, users, and policy makers). | 16,17 |
| Excerpt |  | *In a systematic review of the evidence, we identified a range of evidence-based options to support people with OUD who are incarcerated or recently released from incarceration in the U.S. In reviewed studies, MOUD had significant beneficial impacts on outcomes when treatment was initiated early in criminal justice system involvement and maintained throughout incarceration. While several interventions did integrate social determinants components, these were included in only a minority of interventions reviewed. Results of studies presented in this review is consistent with the current evidence-base regarding MOUD and incarceration, and SDOH as a potential barrier to good health outcomes post-release. However, this review reveals that a gap at the intersection of MOUD, incarceration, and SDOH persists. There is a substantial opportunity to incorporate SDOH into interventions to support the health and well-being of critically at-risk populations who are incarcerated or have been recently released.* |  |
| Limitations | 25 | Discuss limitations at study and outcome level (e.g., risk of bias), and at review-level (e.g., incomplete retrieval of identified research, reporting bias). | 17 |
| Excerpt |  | *This review has several limitations. We may not have identified some pilot programs initiated by county, state, or federal departments of corrections, health departments, or community organizations because we searched only the academic literature…* |  |
| Conclusions | 26 | Provide a general interpretation of the results in the context of other evidence, and implications for future research. | 17, 18 |
| Excerpt |  | *This systematic review of interventions for OUD implemented as part of US criminal justice system involvement synthesized results from several innovative pilot programs and study interventions. The interest in opioid-specific programs and interventions for people involved in the criminal justice system is rising, but more research is needed to understand the key role that addressing SDOH could play in contributing to improved health outcomes. The existing evidence base suggests that medication treatments such as buprenorphine and methadone should administered early in incarceration and continued for the duration of incarceration, particularly for those in prison. Although SDOH were frequently noted as a potential competing priority to engaging in treatment, few interventions to-date have addressed SDOH in the intervention or study design. Those that did include SDOH cited competing priorities as a major determinant of treatment initiation and adherence. Through individual-level interventions or building strong cross-sector collaborations, future interventions for incarcerated people with OUD should integrate medication treatments with interventions to address social determinants of health.* |  |
| **FUNDING** | | |  |
| Funding | 27 | Describe sources of funding for the systematic review and other support (e.g., supply of data); role of funders for the systematic review. | NA |

*From:*  Moher D, Liberati A, Tetzlaff J, Altman DG, The PRISMA Group (2009). Preferred Reporting Items for Systematic Reviews and Meta-Analyses: The PRISMA Statement. PLoS Med 6(7): e1000097. doi:10.1371/journal.pmed1000097

For more information, visit: **www.prisma-statement.org**.

Page 2 of 2
